# Plastic Responses to Elevated Temperature in Low and High Elevation Populations of Three Grassland Species

**DOI:** 10.1371/journal.pone.0098677

**Published:** 2014-06-05

**Authors:** Esther R. Frei, Jaboury Ghazoul, Andrea R. Pluess

**Affiliations:** Department of Environmental Systems Science, Ecosystem Management, ETH Zurich, Zurich, Switzerland; DOE Pacific Northwest National Laboratory, United States of America

## Abstract

Local persistence of plant species in the face of climate change is largely mediated by genetic adaptation and phenotypic plasticity. In species with a wide altitudinal range, population responses to global warming are likely to differ at contrasting elevations. In controlled climate chambers, we investigated the responses of low and high elevation populations (1200 and 1800 m a.s.l.) of three nutrient-poor grassland species, *Trifolium montanum*, *Ranunculus bulbosus,* and *Briza media*, to ambient and elevated temperature. We measured growth-related, reproductive and phenological traits, evaluated differences in trait plasticity and examined whether trait values or plasticities were positively related to approximate fitness and thus under selection. Elevated temperature induced plastic responses in several growth-related traits of all three species. Although flowering phenology was advanced in *T. montanum* and *R. bulbosus*, number of flowers and reproductive allocation were not increased under elevated temperature. Plasticity differed between low and high elevation populations only in leaf traits of *T. montanum* and *B. media*. Some growth-related and phenological traits were under selection. Moreover, plasticities were not correlated with approximate fitness indicating selectively neutral plastic responses to elevated temperature. The observed plasticity in growth-related and phenological traits, albeit variable among species, suggests that plasticity is an important mechanism in mediating plant responses to elevated temperature. However, the capacity of species to respond to climate change through phenotypic plasticity is limited suggesting that the species additionally need evolutionary adaptation to adjust to climate change. The observed selection on several growth-related and phenological traits indicates that the study species have the potential for future evolution in the context of a warming climate.

## Introduction

Global annual mean temperature has risen by approximately 0.85°C over the past century and climate scenarios project a further warming of 2 to 4°C for the 21st century [1]. This rapid temperature increase and the concomitant alterations of other environmental factors, such as higher levels of atmospheric CO_2_ and changing precipitation regimes, will drastically alter living conditions for plant and animal species across the globe and affect individuals, populations and communities [2]–[5]. Temperature is one of the main factors determining plant physiology and performance, and as a consequence, strongly influences the geographic distribution of plants [6], [7]. Therefore, climate warming has caused latitudinal and altitudinal range shifts of many plant species worldwide and further shifts are expected [2], [3]. Along elevation gradients, a considerable increase in number of plant species on alpine summits indicates an upward shift of species’ ranges [8]–[12]. However, the migration potential might be inferior to the rapid rate of current climate change, which is expected to render many species unable to track the climate they are currently adapted to [13], [14]. Continuing habitat loss and fragmentation further exacerbate migration by impeding gene flow [15], [16] and threaten the persistence of many species [17], [18].

Alternatively to shifts in abundance and distribution, plants may persist in a changing climate through evolutionary adaptation and phenotypic plasticity [19]–[22]. Several studies provide evidence of climate driven population differentiation [23]–[26]. Thus, temperature and climate warming might be strong selective agents leading to the adaptive evolution of key plant traits [27], [28]. In contrast to evolutionary adaptation, which requires several generations, current phenotypic plasticity in plants, the ability of a genotype to change its phenotype in response to different environments [29], allows short-term responses to rapid warming [21], [30], [31]. Among the best documented plastic responses to climate change are shifts in phenology observed in many species across the globe [2], [3], [32]. Although these meta-analyses are mainly based on genetically unstructured samples, which could not disentangle genetic and plastic changes, studies in phenological garden networks on structured samples show similar results [33], [34]. Besides plastic shifts in phenology, several studies have documented morphological and physiological trait plasticity to temperature changes, reviewed in [35], and specifically plastic responses to elevated temperature, such as decreased seed dormancy [36], [37] or reduced seed longevity [38].

Adaptive plasticity is the ability of an individual genotype to express phenotypes, which enhance fitness in response to variation in selection [21], [31], [39]. This is likely facilitating species persistence under climate change and assisting rapid evolutionary adaptation [40]–[44]. While numerous studies reported adaptive plasticity to different environmental conditions, such as shade [45], drought [46], [47] and flooding [48], empirical evidence for adaptive plastic responses to climate change is still scarce, but see [49], [50]. The capacity for plastic responses is supposed to vary due to different physiological thresholds, such as temperature and precipitation tolerance. Consequently, trait plasticity generally differs among species, and might even vary among populations from contrasting habitats across a species’ range [50], [51]. Higher trait plasticity would be an advantage for plants experiencing greater spatial and temporal habitat heterogeneity because it allows them to maximize their fitness under different environmental conditions [39], [52], [53].

Mountain ecosystems are characterised by steep climate gradients and by increased environmental variation over short spatial and temporal scales at high as compared to nearby low elevation sites [54]. Because of the greater habitat heterogeneity we hypothesize that high elevation plants exhibit greater trait plasticity than their low elevation congeners. Varying climate conditions along altitudinal gradients might also result in different selection pressures that shape intraspecific trait adaptation to elevation. Little is known about how the adaptive potential of plants and their ability to respond to climate change through phenotypic plasticity shape phenotypic variation along altitudinal gradients. Transplant experiments in the Pyrenees and the Australian Alps revealed genetic differentiation but also trait plasticity [55], [56]. In the Swiss Alps Frei et al. [57] described plasticity in reproductive phenology as an important plant response to transplantation to warmer low elevation sites. However, since these experiments were performed along natural elevation gradients, they were not able to separate the effects of temperature and warming from the influence of concomitant climate factors.

In this study we grew plants in controlled climate chambers and investigated responses of low (1200 m a.s.l.) and high (1800 m a.s.l.) elevation populations of three montane grassland species, *Trifolium montanum* L., *Ranunculus bulbosus* L. and *Briza media* L. to experimental warming. Plants were grown under elevated temperature, corresponding to predicted future conditions under climate change, and in control treatments corresponding to current ambient conditions at the plant origin whereas all other environmental parameters were kept identical. We measured growth-related, reproductive and phenological traits of the experimental plants and assessed differences in trait plasticity of low and high elevation populations. Using phenotypic selection analyses [58], we examined whether trait values and plasticities are under selection. Specifically, we addressed the following questions: (i) How are plant traits and fitness affected by elevated temperature? (ii) Does the degree of trait plasticity vary between low and high elevation populations? (iii) Are traits under selection? Does the strength or direction of selection differ between ambient and elevated temperature conditions? (iv) Does variation in temperature impose selection on trait plasticity? Studying current levels of genetic differentiation and variation in trait plasticity in response to elevated temperature will lead to a better understanding of future plant responses to predicted climate change.

## Materials and Methods

### Study Species

The common perennial species *Trifolium montanum* L., *Ranunculus bulbosus* L. and *Briza media* L. were selected for this study based on their co-occurrence in semi-dry nutrient-poor calcareous grasslands and their similar altitudinal range from 400 to 2000 m a.s.l [59]. The three species differ in phenology, pollination syndromes and reproduction: *Trifolium montanum* is an insect-pollinated late-flowering herb, *R. bulbosus* an insect-pollinated early-flowering herb and *B. media* is a wind-pollinated grass.

The leguminous *Trifolium montanum*, which forms a symbiosis with soil rhizobia, produces 1–5 flowering shoots from June to July. A shoot bears 1–6 inflorescences with c. 150 white flowers each, which are pollinated by bumblebees and bees. Seeds are small (c. 0.7 mg), but dispersal distance is very limited as they are primarily gravity-dispersed [60].

*Ranunculus bulbosus* produces a corm that serves as nutrient storage and as summer perennating organ [61]. In late winter, *R. bulbosus* begins to mobilize corm reserves to add new leaves to the overwintering rosette and starts to form a new corm. From late April to July, the plant produces one or more flower stalks of c. 30 cm height bearing 8–15 yellow flowers [62]. The seeds lack dispersal aids and are therefore expected to be mainly gravity dispersed. After a period of aestivation, a new rosette is formed in early autumn [63], [64].

*Briza media* reproduces clonally and sexually with each ramet potentially forming one flower stalk [65]. The flowers emerge from June to July, have large feathery stigmas, and mature into indehiscent fruits that disperse next to the mother plant mainly by gravity [65]. Long-distance seed dispersal by grazing animals has also been observed [66].

### Plant Material

In summer 2008, seeds were collected in seven to thirteen population pairs per species across the Swiss Alps (Table S1). For seed sampling, permits were obtained from the managers of the respective sites. Each population pair consisted of one population in the centre of the species range at c. 1200 m a.s.l. (1095–1275 m a.s.l.) and another close to the upper range limit at c. 1800 m a.s.l. (1720–1860 m a.s.l.), hereafter referred to as low and high elevation populations respectively. The vertical distance of 600 m between these elevations approximates a difference in annual mean temperature of 4°C [54] corresponding to the expected temperature rise of a typical climate change scenario for the year 2100 [1]. The horizontal distance between the two populations of a pair was 1–18 kilometres. Seeds were sampled from at least ten maternal plants in each population, air-dried and stored in paper bags at 4°C.

In spring 2009, seeds were germinated in a greenhouse. After four to six weeks, seedlings were planted into individual pots (800 cm^3^), filled with a 3∶2 mixture of nutrient-poor commercial soil and sand. Pot positions were randomized weekly. From October 2009 to April 2010, the plants were overwintered outdoors at Davos (1500 m a.s.l.) to support vernalisation processes.

### Climate Chamber Experiment

In May 2010, a controlled climate chamber experiment was established to test the responses of plants to current ambient temperature, corresponding to the temperature at their altitude of origin, and to elevated temperature, corresponding to 4°C higher temperature as compared to current temperature at their altitude of origin. The lowest temperature treatment (hereafter ‘T_min_’) was ambient temperature for plants of high elevation origin (1800 m a.s.l.). Elevated temperature for high elevation plants was equivalent to ambient temperature for plants of low elevation origin (1200 m a.s.l.), and was designated as ‘T’. Elevated temperature for low elevation plants was denoted as ‘T_plus_’ and corresponded to ambient temperature at 600 m a.s.l. Each temperature treatment was replicated in two climate chambers. To separate temperature from other climate effects, only temperature was varied in this experiment while humidity and illumination treatments were kept the same in all chambers. Temperature, humidity and light regime followed both a daily as well as a seasonal cycle. To simulate the daily temperature cycle with lower temperature, higher humidity and dark conditions at night and opposed conditions during the day, seven step cycles were programmed according to climate station measurements [67]. For example in July, temperatures, relative humidity and light were varied diurnally between 5°C/80%/0 klx as the night’s minimum and 18°C/48%/21 klx as the daily maximum (Table 1). To account for seasonal changes of the climate regime, program cycles were adapted monthly.

**Table 1:** Daily cyles of the climate chamber program for the three temperature treatments T_min_, T and T_plus_ reflecting July outdoor temperatures at 1800 m, 1200 m and 600 m a.s.l.

Interval	Running time (h)	Temperature (°C)			Relative humidity (%)	Illumination (klx)
		T_min_	T	T_plus_		
1	2	8	12	16	72	0
2	3	6	10	14	81	0
3	5	5	9	13	84	0
4	6	18	22	26	55	21
5	2	19	23	27	51	21
6	2	18	22	26	51	16
7	4	10	14	18	63	1

Parameters were changed gradually to reach the set value at the end of the running time of each interval. Relative humidity and light regime were kept the same in all temperature treatments.

The plants of each species and population were assigned equally to ambient and elevated temperature treatments and to both climate chambers per treatment. They were rotated weekly within each chamber and monthly between the two chambers of the same treatment. The experiment included 174 plants from ten population pairs of *T. montanum*, 88 plants from seven population pairs of *R. bulbosus* and 312 plants from 13 population pairs of *B.* *media*.

### Assessment of Plant Traits

In May 2010 and at the end of the growing season (October 2010), number of leaves (respectively ramets for *B. media*) and length of the longest leaf of each plant were measured. To obtain a measure for growth rate, the increment of number of leaves (respectively ramets for *B. media*) was calculated. In September 2010, one mature leaf per individual plant was sampled to assess specific leaf area (SLA). Immediately after sampling, the leaf blades were scanned (HP AllInOne colour Scanner, Hewlett-Packard GmbH, Dübendorf, Schweiz) and their areas were determined with lamina version 1.0.2 [68]. The scanned leaf blades were oven-dried at 60°C for 48 h and weighed. To determine SLA, we divided the leaf area by the dry weight of the leaf blade [69].

From May to July 2010, 75% of *T. montanum* and 84% of *R.* *bulbosus* individuals flowered. Due to the low flowering rate in *B. media* (8%), reproductive and phenological traits were only assessed for the two herbs. The phenological development was monitored twice a week to record the Julian Day (JD) of appearance of the first flower bud and the first open flower of each individual. In August, flowers of *R. bulbosus* and inflorescences of *T. montanum* were counted and the reproductive biomass (i.e. flower stalks and flowers) was harvested.

In October 2010, all plants were harvested and oven-dried. Above-ground (i.e. reproductive organs and leaves) and below-ground biomass of *T. montanum* and *R. bulbosus* were measured separately. Subsequently, biomass partitioning (i.e. above-ground biomass as a proportion of total biomass) and reproductive allocation (i.e. the proportion of reproductive biomass on total above-ground biomass) were calculated. In *B. media*, only above-ground biomass was assessed because we were not able to remove all soil sticking to the fine root system without considerable loss of root biomass.

### Statistical Analyses

We first tested for effects of elevated temperature and genetic differentiation on phenotypic trait variation. Then, we conducted two sets of selection gradient analyses where we tested for selection on plant traits as well as for selection on trait plasticities in response to elevated temperature. All statistical analyses were performed with the statistical software R [70]. The residuals were checked for deviations from the model assumptions and the data was transformed if necessary [71]. To account for multiple tests we applied a Bonferroni correction within species whenever applicable.

We performed nested mixed-model analyses of covariance (Ancova) for each species separately to analyse the effects of elevated temperature and altitude of origin. The model consisted of the two fixed factors ‘altitude of origin’ and ‘temperature treatment’, the random factor ‘population’ and the two interaction terms ‘temperature treatment × altitude of origin’ and ‘temperature treatment × population’. A significant ‘altitude of origin’ effect (G_Alt_) indicates genetic differentiation between low and high elevation populations. A significant ‘temperature treatment’ effect (E_Temp_) indicates trait variation due to different environmental conditions i.e. phenotypic plasticity in a measured trait. A significant ‘temperature treatment × altitude of origin’ interaction (G_Alt_×E_Temp_) indicates differences in plasticity between the altitudes of origin. The ‘altitude of origin’ was tested on the population level and the ‘temperature treatment’ on the interaction of ‘temperature treatment × population’ while all other terms were tested on the residuals. Besides direct responses to the environment, our measurements of plasticity might also be influenced by ontogenetic drift since the analyses did not account for plant size [72]. By including initial plant size (length of the longest leaf) as a covariate in the models we account for possible maternal effects [73].

We conducted phenotypic selection analyses on trait values in each temperature treatment to assess whether traits were under selection [58], [74]. Selection gradients were calculated based on regressions of relative fitness, measured as total biomass (*T. montanum* and *R. bulbosus*) and above ground biomass (*B. media*), on standardized trait values of individual plants. A trait was under positive selection if trait values were positively correlated with genotype fitness. To test whether selection differed between treatments, we calculated Ancovas of relative fitness in which we included the trait as a covariate and its interaction with the temperature treatment [48].

Phenotypic selection analyses on trait plasticities were performed to test whether trait plasticity itself was under selection, i.e. if plasticity was positively correlated with overall fitness, indicating adaptive plasticity. Selection gradients on trait plasticity were determined by regressing average fitness of a population on population trait values averaged over both treatments (i.e. elevation of the reaction norm) and on population values of plasticity (i.e. steepness of the reaction norm) [75], [76]. The elevation of the reaction norm was included to disentangle the fitness effect of the average value from the fitness effect of plasticity [77]. As proxies for fitness, we used number of inflorescences (*T. montanum* and *R. bulbosus*), total biomass (*T. montanum* and *R. bulbosus*) and above ground biomass (*B. media*). For both sets of selection gradient analyses, regression coefficients were standardized by expressing them in units of 1 SD to allow comparisons between traits and fitness measures.

## Results

### Environmental and Genetic Effects on Plant Variation

The model analyses allowed us to explain trait variation due to environmental effects of elevated temperature (E_Temp_) and genetic differentiation (G_Alt_), as well as their interactions (G_Alt_×E_Temp_). The random term population nested within altitude of origin had essentially no influence on trait variation (*P*>0.165) except for a difference in the proportion of above-ground biomass in *T. montanum* and *B. media* (*P*<0.001), as well as a tendency for different budding starts in *R. bulbosus* (*P = *0.056; Tables 2 and 3). The covariate had a significant influence on the majority of traits (Tables 2 and 3). However, omitting the covariate did not change the results qualitatively (details not shown).

**Table 2:** Nested mixed-model Ancova for growth-related traits of *Trifolium montanum*, *Ranunculus bulbosus* and *Briza media* grown under ambient and elevated temperature.

		Biomass		Leaf length		Growth rate		SLA			Above-ground/total biomass	
	d.f.	MS	*F*	MS	*F*	MS	*F*	MS	*F*		MS	*F*
*T. montanum*												
Initial plant size	1	19.31	76.26***	167.18	16.77***	4.74	14.36**	105822.00	2.99		1.23	16.52***
Temp	1	7.07	27.91**	108.63	10.89**	4.16	12.59*	157334.00	4.45		0.73	9.83(*)
Orig	1	15.24	60.18***	198.74	19.93**	0.07	0.22	76416.00	2.16		0.49	6.57
Pop[Orig]	18	0.38	1.52	11.47	1.15	0.50	1.53	47855.00	1.35		0.28	3.75***
Temp × Orig	1	0.49	1.95	243.42	24.41***	0.33	0.99	38816.00	1.10		0.06	0.84
Temp × Pop	18	0.32	1.25	7.21	0.72	0.35	1.06	43784.00	1.24		0.08	1.04
Residuals	128	0.25		9.97		0.33		35383.00			0.07	
*R. bulbosus*												
Initial plant size	1	15.99	22.61***	0.26	0.21	394.70	12.96**	6230.20	5.16		0.00	0.13
Temp	1	1.06	1.50	10.56	8.49(*)	378.12	12.41*	2107.50	1.75		0.00	0.11
Orig	1	0.05	0.07	26.08	20.96**	504.64	16.56	30617.20	25.38***		0.03	0.77
Pop[Orig]	12	0.88	1.24	1.18	0.95	66.72	2.19	863.10	0.72		0.04	1.31
Temp × Orig	1	0.49	0.70	0.22	0.17	18.98	0.62	10155.70	8.42(*)		0.01	0.28
Temp × Pop	12	0.88	1.25	0.94	0.76	21.58	0.71	1267.10	1.05		0.02	0.46
Residuals	47–59	0.71		1.24		30.47		1206.40			0.03	
*B. media*												
Initial plant size	1	4.33	86.02***	54.40	14.45***	9.19	0.95	0.22	5.30(*)			
Temp	1	1.74	34.68***	14.84	3.94*	132.64	13.71*	0.04	0.87			
Orig	1	1.29	25.66*	0.05	0.01	24.62	2.54	0.09	2.14			
Pop[Orig]	24	0.17	3.36***	5.56	1.48	15.46	1.60	0.04	0.89			
Temp × Orig	1	0.11	2.13	333.25	88.50***	1.62	0.17	1.31	31.99***			
Temp × Pop	24	0.03	0.67	1.91	0.51	12.45	1.29	0.04	0.99			
Residuals	259	0.05		3.77		9.68		0.04				

d.f., degrees of freedom; MS, mean squares; *F*, *F*-values; Initial plant size, covariate; Temp, temperature treatment; Orig, altitude of population origin; Pop, population. Residual d.f. vary per trait due to missingness. Bonferroni-corrected significance levels within species are indicated by asterisks: ***P_Bonf_ <0.001, **P_Bonf_ <0.01, *P_Bonf_ <0.05, (*)*P*_Bonf_<0.08.

**Table 3:** Nested mixed-model Ancova for reproductive and phenological traits of *Trifolium montanum* and *Ranunculus bulbosus* grown under ambient and elevated temperature.

		Number of flowers		Reproductive allocation		Budding start		Flowering start	
	d.f.	MS	*F*	MS	*F*	MS	*F*	MS	*F*
*T. montanum*									
Initial plant size	1	64.76	47.07***	1.96	42.38***	24721.90	30.61***	17.43	36.37***
Temp	1	0.25	0.18	0.01	0.17	5641.00	6.98**	11.24	23.46***
Orig	1	6.33	4.60	0.12	2.55	7049.00	8.73*	10.79	22.51**
Pop[Orig]	18	2.49	3	0.09	1.84	680.80	0.84	0.58	1.22
Temp × Orig	1	8.53	6.20	0.02	0.50	0.10	0.00	1.00	2.09
Temp × Pop	17–18	0.96	0.70	0.03	0.71	340.00	0.42	0.23	0.47
Residuals	77–128	1.38		0.05		807.80		0.48	
*R. bulbosus*									
Initial plant size	1	18.19	22.86***	0.12	8.82*	3312.60	23.61***	3925.90	26.58***
Temp	1	3.08	3.87	0.02	1.73	1188.00	8.47***	2238.00	15.15***
Orig	1	1.88	2.36	0.14	10.12	627.10	4.47	2197.50	14.88(*)
Pop[Orig]	12	0.97	1.23	0.02	1.27	391.80	2.79(*)	219.70	1.49
Temp × Orig	1	0.69	0.86	0.01	0.43	16.90	0.12	3.30	0.02
Temp × Pop	12	0.77	0.97	0.02	1.67	30.30	0.22	29.00	0.20
Residuals	45–59	0.80		0.01		140.30		147.70	

d.f., degrees of freedom; MS, mean squares; *F*, *F*-values; Initial plant size, covariate; Temp, temperature treatment; Orig, altitude of population origin; Pop, population. Residual d.f. vary per trait due to missingness. Bonferroni-corrected significance levels within species are indicated by asterisks: ***P_Bonf_ <0.001, **P_Bonf_ <0.01, *P_Bonf_ <0.05, (*)*P*_Bonf_<0.08.

#### Plant responses to elevated temperature

**Figure 1:**
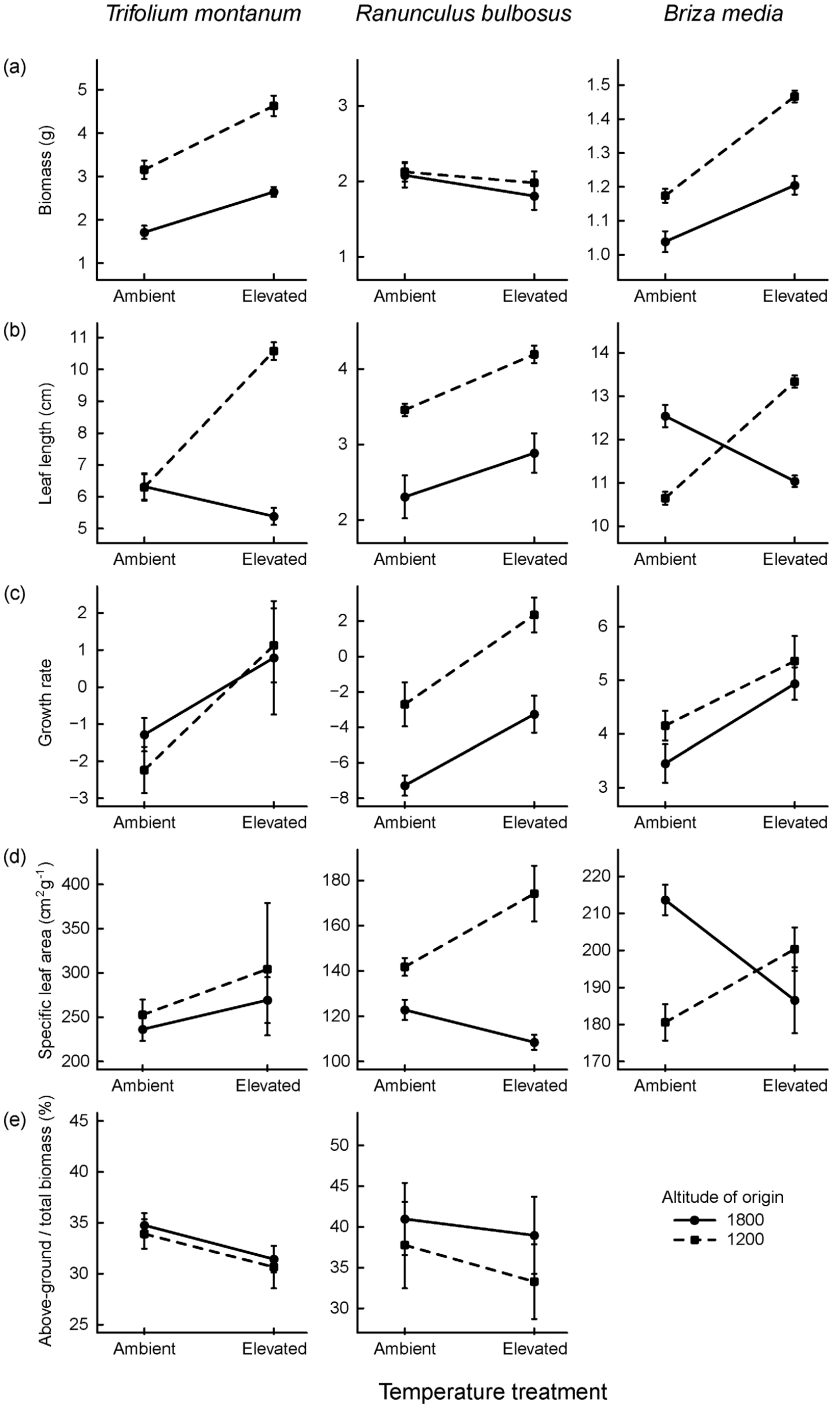
Population average reaction norms for growth-related traits of *Trifolium montanum*, *Ranunculus bulbosus* and *Briza media* in response to ambient and elevated temperature in a climate chamber experiment. (a) total biomass (above-ground biomass for *Briza media*), (b) leaf length, (c) growth rate, (d) specific leaf area and (e) proportion of above ground biomass. Solid lines indicate high elevation plants (1800 m a.s.l.) and dashed lines low elevation plants (1200 m a.s.l.). Error bars indicate standard errors of trait means.

**Figure 2:**
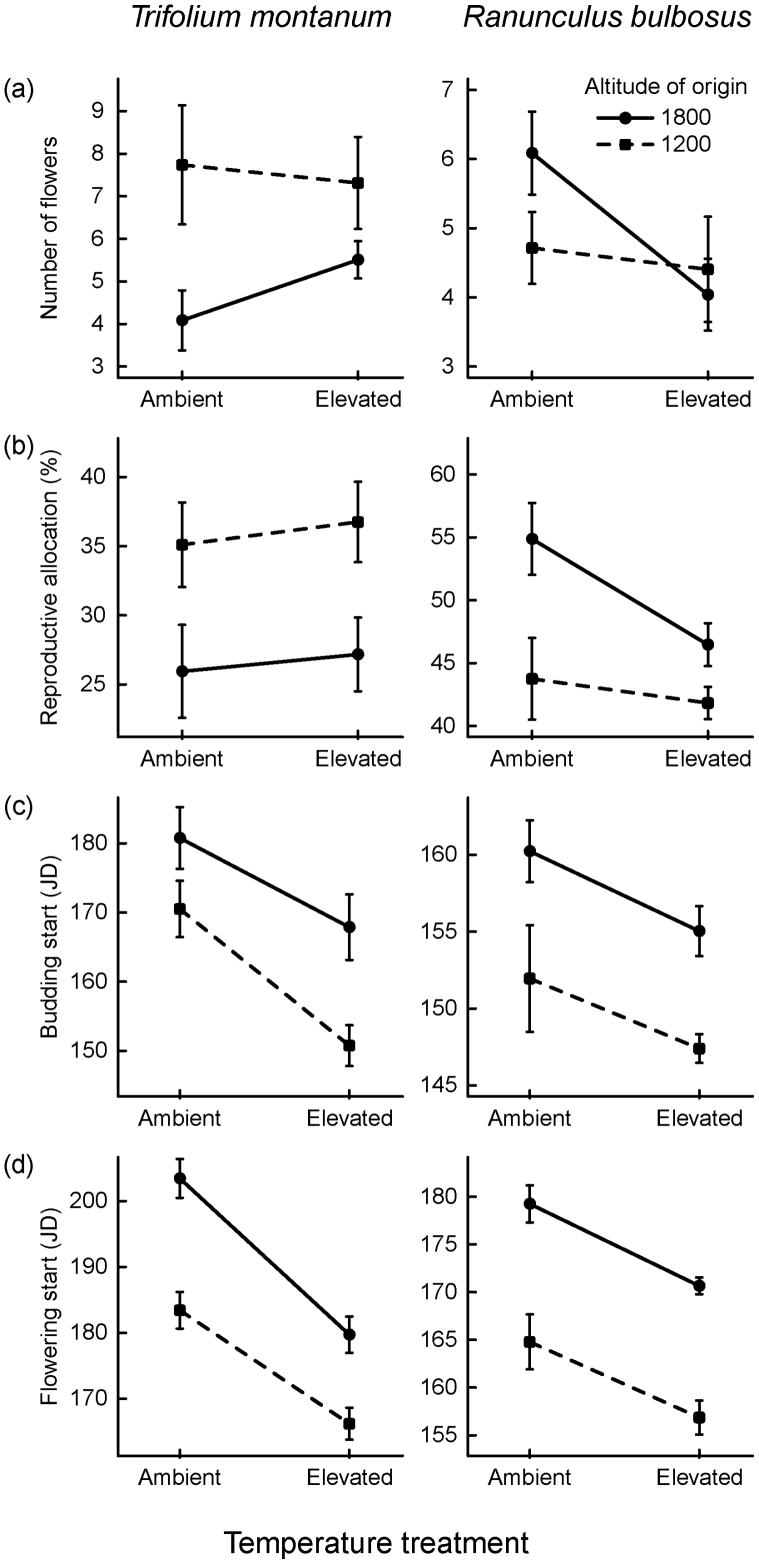
Population average reaction norms for reproductive and phenological traits of *Trifolium montanum* and *Ranunculus bulbosus* in response to ambient and elevated temperature in a climate chamber experiment. (a) number of flowers, (b) reproductive allocation, (c) budding start and (d) flowering start. Solid lines indicate high elevation plants (1800 m a.s.l.) and dashed lines low elevation plants (1200 m a.s.l.). Error bars indicate standard errors of trait means.

*Trifolium montanum* responded to elevated temperature with an increase in total biomass (E_Temp_: *P* = 0.002; Tables 2 and S2; Fig. 1) and growth rate (E_Temp_: *P = *0.026), but a slightly reduced proportion of above-ground biomass (E_Temp_: *P* = 0.059). SLA was unaffected by temperature (E_Temp_: *P* = 0.668). Flower bud initiation and anthesis were advanced under elevated temperature (E_Temp_: *P*<0.007; Tables 3 and S3; Fig. 2) whereas number of flowers and reproductive allocation showed no temperature-induced plasticity (E_Temp_: *P*>0.9).

In *R. bulbosus*, growth rate increased (E_Temp_: *P* = 0.011; Table 2; Fig. 1) and leaf length tended to increase (E_Temp_: *P* = 0.053) under elevated temperature whereas total biomass, SLA and proportion of above ground biomass were not affected by temperature (E_Temp_: *P*>0.9). Flowering phenology was advanced under elevated temperature with buds and flowers appearing earlier (E_Temp_: *P*<0.001; Table 3; Fig. 2) whereas number of flowers and reproductive allocation were unaffected by temperature (E_Temp_: *P*>0.139).

In *B. media*, leaf length, above-ground biomass and growth rate increased under elevated temperature (E_Temp_: *P*<0.041; Table 2; Fig. 1) and SLA was unaffected by temperature (E_Temp_: *P*>0.9).

#### Genetic differentiation in traits and plasticity

In *T. montanum*, plants of low elevation origin produced almost twice as much biomass (G_Alt_: *P*<0.001; Table 2, Fig. 1) and longer leaves (G_Alt_: *P* = 0.005) than high elevation plants whereas growth rate, SLA and the proportion of above-ground biomass did not differ between altitudes of origin (*P*>0.9). Budding and flowering started earlier in low as compared to high elevation plants (G_Alt_: *P*<0.043; Table 3, Fig. 2). Number of flowers and reproductive allocation did not differ between low and high elevation plants (G_Alt_: *P*>0.9). Furthermore, plasticity differed between altitudes of origin only in leaf length, where trait means almost doubled in low elevation plants but remained nearly unchanged in high elevation plants under elevated temperature (G_Alt_×E_Temp_: *P*<0.001; Table 2, Fig. 1). Plasticity of total biomass, growth rate, SLA and proportion of above-ground biomass as well as reproductive and phenological traits did not differ between low and high elevation populations (G_Alt_×E_Temp_: *P*>0.1; Tables 2 and 3; Figs. 1 and 2).

In *R. bulbosus*, low elevation plants produced longer leaves (G_Alt_: *P* = 0.005; Table 2; Fig. 1) and larger SLA (G_Alt_: *P*<0.001) than high elevation plants whereas total biomass, growth rates and the proportion of above-ground biomass did not differ between low and high elevation plants (G_Alt_: *P*>0.158). Low elevation plants tended to flower earlier than high elevation plants (G_Alt_: *P* = 0.074; Table 3; Fig. 2). Number of flowers, reproductive allocation and budding dates did not differ between low and high elevation plants (G_Alt_: *P*>0.139). Furthermore, there were no significant differences in plasticity between low and high elevation populations neither in growth-related traits (G_Alt_×E_Temp_: *P*>0.07; Table 2; Fig. 1) nor in reproductive and phenological traits (G_Alt_×E_Temp_: *P*>0.9; Table 3; Fig. 2).

In *B. media*, low elevation plants produced more above ground biomass than high elevation plants (G_Alt_: *P = *0.043; Table 2; Fig. 1). Leaf length, growth rate and SLA did not differ between low and high elevation plants (G_Alt_: *P*>0.534). Furthermore, plasticity of leaf traits differed between altitudes of origin: leaf length and SLA increased in low elevation but decreased in high elevation plants under elevated temperature (G_Alt_×E_Temp_: *P*<0.001; Table 2; Fig. 1). Plasticity of above ground biomass and growth rate did not differ between altitudes of origin (G_Alt_×E_Temp_: *P*>0.584).

### Selection Gradient Analyses on Traits and Plasticities

The selection gradient analyses on trait values in *T. montanum* revealed negative selection gradients for flower bud initiation and the appearance of the first flower averaged over both temperature treatments, which was reflected in a significant effect of these traits on fitness measured as total biomass (*P*<0.008; Table 4). In *R. bulbosus*, the selection analyses indicated a negative effect of SLA on fitness measured as total biomass (*P* = 0.003; Table 4). In *B. media*, there was selection for higher growth rates averaged over both treatments (*P*<0.001). Selection gradient analyses on trait plasticities revealed no direct selection for plasticity in response to temperature in all traits and species (*P*>0.148; Table 5).

**Table 4:** Selection gradients of growth-related and phenological traits of *Trifolium montanum*, *Ranunculus bulbosus* and *Briza media* under ambient and elevated temperature and F-values of ANCOVA testing for overall selection (Cov) and differences in selection between temperature treatments (Cov × Temp).

	Selection gradients		F-Values of ANCOVA	
	ambient	elevated	Cov	Cov × Temp
*T. montanum*				
Growth rate	−0.320	0.009	0.15	4.57
SLA	−0.332	0.009	0.85	0.17
Budding start	−0.543	−0.170	10.07**	0.80
Flowering start	−0.804	−0.297	19.58***	1.40
*R. bulbosus*				
Growth rate	−0.279	−0.071	4.88	1.35
SLA	−0.365	−0.309	12.68**	0.70
Budding start	−0.119	−0.145	0.94	0.04
Flowering start	−0.236	−0.309	4.50	0.23
*B. media*				
Growth rate	0.044	0.079	20.70***	0.02
SLA	−0.040	0.025	0.38	3.38

Selection gradients are expressed as standardized regression coefficients after regression of the fitness measures total biomass (*T. montanum*, *R. bulbosus*) and above ground biomass (*B. media*) on the respective trait. All calculations are based on individual plant values. Bonferroni-corrected significance levels are indicated by asterisks: ***P<0.001, **P<0.01, *P<0.05.

**Table 5:** Selection gradients of trait plasticities in response to temperature in *Trifolium montanum*, *Ranunculus bulbosus* and *Briza media*.

	*T. montanum*		*R. bulbosus*		*B. media*
	Total biomass	Number of flowers	Total biomass	Number of flowers	Above ground biomass
Growth rate	0.500	0.455	0.064	0.258	0.366
SLA	0.423	0.541	−1.380	−0.418	−0.577
Above-ground/total biomass	−0.497	−0.189	−1.334	−0.450	–
Budding start	0.289	0.480	−0.171	0.315	–
Flowering start	0.307	0.380	0.039	1.156	–

Selection gradients are expressed as standardized regression coefficients. All values are based on population means. Fitness measures are total biomass and number of flowers (*T. montanum*, *R. bulbosus*) and above ground biomass (*B. media*). Non of the selection gradients was significant.

## Discussion

In the present study, we evaluated the effects of elevated temperature on phenotypic variation of the three perennial grassland species *T. montanum*, *R. bulbosus* and *B. media*. We investigated if trait plasticity varies between low and high elevation populations and if trait values and plasticities were under selection.

### Plastic Responses to Elevated Temperature

Positive warming effects on several growth-related traits in all three species (Table 2) indicate that plant growth is constrained by low temperature conditions, which is common in temperate species [6]. In arctic and temperate zones plant species spend the majority of their life at mean temperatures below the growth optimum [78]. Thus, a moderate warming could enhance plant performance. Meta-analyses of in-situ warming experiments with arctic and alpine tundra species revealed biome-wide trends of increased vegetative growth, albeit variable among species [79], [80]. The variable responses to elevated temperature of the three study species might be associated with differences in characteristics of below-ground organs. Besides the positive responses in the other growth-related traits, the nitrogen-fixing *T. montanum* showed a slight decrease in the proportion of above ground biomass. The observed greater investment in roots under elevated temperature is contrary to the general trend that allocation to root biomass increases with decreasing temperature at higher elevation [54]. However, it is in line with the findings of Roughley and Dart [81] who described a positive effect of soil temperature on the formation of rhizobia in *Trifolium subterraneum*. The increased number of rhizobia enhances nitrogen availability, which stimulates root growth. *Fabaceae*, such as *T. montanum*, might therefore gain a competitive advantage over non-nitrogen-fixing species under warmer climate conditions. Competition experiments with elevated CO_2_, which has a similar positive effect on nitrogen fixation as elevated temperature, provide evidence for such a competitive advantage, reviewed in [82]. The less pronounced warming effect on growth-related traits in *R. bulbosus* could be related to the species-specific life-form. The nutrient-storing corm might allow a temperature independent formation of new above ground tissue after the period of summer aestivation and buffer against short-term climatic fluctuations as shown for *R. nivalis* [83].

Preformation of flower buds might lead to a time lag in the reproductive response to warming potentially explaining why the experimental warming did not affect reproductive traits of *T. montanum* and *R. bulbosus* (Table 3). This phenomenon is relatively common among temperate herbaceous perennials [84] and has been documented for tundra species in in-situ warming experiments [79]. Thus, it might have required at least two growing seasons to provide a strong estimate of the effects of increased temperature on reproduction of the perennial study species. Although these species did not respond plastically to elevated temperature, they are likely to be affected by changes in other environmental factors (e.g. reduced precipitation and higher evapotranspiration) co-occurring in natural systems. Alternatively, the lack of reproductive responses could indicate that reproductive traits are genetically fixed suggesting that these species need to adjust to climate change through the relatively slow process of evolutionary adaptation [20], [85]. In the context of rapid climate change, this process might be too slow to secure plant persistence [86]–[88]. Evidence for evolutionary adaptation to climate change is still scarce, and rapid evolution has been documented mainly in fast-growing annuals [27], [89] but also in perennials [90].

Reproductive phenology of *T. montanum* and *R. bulbosus* was advanced under elevated temperature (Table 3) confirming the findings of other studies that these traits respond plastically to temperature, e.g. [2], [5], [79], [91]–[95]. Moreover, our findings are in line with the advanced reproductive phenology of the same species grown in a common garden transplant experiment [57]. The advanced spring phenology facilitates setting flowers and seeds before the hot and dry midsummer period. It might therefore enhance plant fitness, but also bears the risk of damage by late frost events, especially in high elevation populations [54], [96], [97]. Furthermore, plant-pollinator-interaction models indicate that pollinating insects might not keep up with shifted flowering periods [98], which might impede insect pollination and thereby reduce reproductive success [99], [100].

In summary, low and high elevation populations exhibited plasticity to temperature in reproductive phenology and several growth-related traits. The advanced flowering and the generally enhanced growth, albeit variable among species, indicate the ability of these species to cope with climate warming at least in the short term.

### Genetic Variation in Traits and Differences in Plasticity between Low and High Elevation Populations

We found only little genetic variation among populations within each altitudinal origin (Tables 2 and 3) suggesting small selection differences and high genetic connectivity. Indeed, a previous study on similar populations of the same three species using neutral molecular markers revealed intermediate genetic diversity but only low genetic differentiation among populations and assigned this to extensive historic gene flow [101]. The genetic differentiation between low and high elevation populations in some growth-related and phenological traits (Tables 2 and 3) indicated that selection in the past has acted differently on low and high elevation plants. Alternatively, the observed genetic differences might be due to neutral genetic processes [24], [102]. However, maternal effects were accounted for by including initial plant size as a covariate in the models and genetic drift is unlikely to have acted in the same direction on all sampled populations since seeds were sampled from multiple population pairs distributed over the Swiss Alps.

In contrast to the hypothesis of greater plasticity at more heterogeneous high elevation sites, plasticity of leaf length was reduced in high elevation populations of *T. montanum* (Table 2; Fig. 1). Similarly, a study with seedlings of European deciduous tree species found that high elevation provenances exhibited less temperature-induced plasticity in growth and leaf phenological traits [103], [104]. The authors argued that low plasticity at high elevations was a result of different directional selection for reduced temperature sensitivity and a stronger influence of photoperiodism, which may reduce the risk of damage by unpredictable late spring frost events [105]. Moreover, the greater plasticity of leaf length in low elevation plants might have been induced by higher levels of competition at these sites [106]. In *B. media*, plastic responses of leaf length and SLA to elevated temperature were positive in plants from warmer low elevations, but negative in plants from colder high elevations (Table 2; Fig. 1). In low elevation plants, elevated temperature induced longer leaves and greater SLA. Thus, the warming seems to have triggered a greater investment in vegetative growth. High elevation plants invested more in clonal propagation as can be concluded from their shorter and thicker leaves as indicated by the smaller leaf length and SLA in combination with an increase in number of ramets (details not shown). In-situ warming experiments showed similar response patterns, reviewed in [79]. The stronger growth response in low arctic plants was related to higher competition in these communities [107] whereas the greater reproductive response of high arctic plants was attributed to increased colonization efforts in these habitats where competition is less important [108].

In summary, plasticity differed between altitudes of origin in only a few traits and there was little genetic differentiation among populations within each altitudinal origin suggesting uniform responses to climate warming. Moreover, the high connectivity together with the moderate genetic differentiation between altitudes of origin might facilitate future adaptation to climate change.

### Selection on Traits and their Plasticities

We found indication for selection on several growth-related and phenological traits (Table 4). In *T. montanum*, the two phenological traits budding and flowering start were under negative selection with respect to biomass: plants with earlier appearing flower buds and flowers ended up with higher biomass at the end of the growing season. Although we did not record leaf phenology, it is likely that the advanced flowering phenology was related to a similar advance in other spring phenophases [32] allowing these plants more time to accumulate biomass. In *B. media*, selection for higher growth rates can be explained by the fact that faster growing individuals acquired more above ground biomass [7].

Although plasticity to temperature was not adaptive in our experiment (Table 5), increased plant size under elevated temperature might eventually lead to higher fitness since flower bud preformation is common in perennials and sexual reproduction is often positively correlated with plant size [109]. Increased growth under warmer temperature might thus be beneficial for plant persistence in the longer term whereas the duration of our experiment might have been too short for observing fitness benefits of plasticity. The magnitude and even the direction of selection on a trait may differ for different components of fitness [74]. This might also have affected the results of our selection analyses and shows the need for more comprehensive fitness measures [110]. Furthermore, it is generally recommended to analyse phenotypic selection on trait means and their plasticities based on genotypic values [111] to avoid environmentally induced covariation [112]. However, our experimental design only allowed us to analyse selection based on individual values or population means, see [113]. This might have resulted in a loss of discriminative power because the observed variation is a combination of phenotypic plasticity and within population genetic variability. Thus, future selection studies at the genotype level are recommended to gain deeper insights into the mechanisms of plasticity in plant populations.

In summary, the absence of selection on phenotypic plasticity indicated that the observed trait plasticity was selectively neutral. Selection on several growth-related and phenological traits suggested that there is potential for future evolution of mean trait values allowing the study species to adapt to elevated temperature provided there is sufficient genetic variation and heritability of the respective traits.

### Conclusions

We investigated phenotypic variation in low and high elevation populations of nutrient-poor grassland species in response to elevated temperature. The three species exhibited trait plasticity with respect to temperature whereas genetic differentiation and differences in plasticity between low and high populations were less important. Plasticity in growth and flowering phenology determines the ability of the study species to respond to elevated temperature by buffering against detrimental effects of rapid climate change and allowing time for evolutionary adaptation. However, the capacity of species to respond to environmental changes through phenotypic plasticity is limited and plasticity alone might not be sufficient to cope with climate change. Thus, plants additionally need to adjust through the relatively slow process of evolutionary adaptation. Selection on several traits suggests that the three species have the potential for evolutionary changes, which might allow them to adapt to a future climate.

## Supporting Information

Table S1 **Sampling locations in the Swiss Alps of the source population pairs of** ***Trifolium montanum*** **(Tm),** ***Ranunculus bulbosus*** **(Rb) and** ***Briza media*** **(Bm).**
(DOCX)

Table S2 **Means and standard errors (SE) grouped by altitude of population origin and temperature treatment for growth-related traits of** ***Trifolium montanum*** **,** ***Ranunculus bulbosus*** **and** ***Briza media*** **.**
(DOCX)

Table S3 **Means and standard errors (SE) grouped by altitude of population origin and temperature treatment for reproductive and phenological traits of** ***Trifolium montanum*** **and** ***Ranunculus bulbosus*** **.**
(DOCX)
