# Supplementary material for: Plastic Responses to Elevated Temperature in Low and High Elevation Populations of Three Grassland Species
Source: PLoS One. 2014 Jun 5;9(6):e98677. doi: 10.1371/journal.pone.0098677 (PMC4046993; doi:10.1371/journal.pone.0098677)
Supplement: Table S1 — Sampling locations in the Swiss Alps of the source population pairs of Trifolium montanum (Tm), Ranunculus bulbosus (Rb) and Briza media (Bm). (DOCX) [file pone.0098677.s001.docx]

Supporting Information for doi:10.1371/journal.pone.0098677

| **Table S1.** Sampling locations of the source population pairs of *Trifolium montanum* (Tm), *Ranunculus bulbosus* (Rb) and *Briza media* (Bm) in the Swiss Alps. | | | | | |
| --- | --- | --- | --- | --- | --- |
| ID | Site name | Species | Longitude  (Swiss Grid) | Latitude  (Swiss Grid) | Altitude (m a.s.l) |
|  |  |  |  |  |  |
| 1 | Bex1200 | Bm, Tm | 573179 | 125487 | 1170 |
| 2 | Bex1800 | Bm, Tm | 577768 | 127268 | 1760 |
| 3 | Brie1200 | Bm, Tm | 636081 | 174239 | 1120 |
| 4 | Brie1800 | Bm, Tm | 635657 | 174788 | 1775 |
| 5 | Chat1200 | Bm, Rb | 576324 | 148377 | 1265 |
| 6 | Chat1200 | Tm | 574805 | 146862 | 1220 |
| 7 | Chat1800 | Rb | 579911 | 142047 | 1740 |
| 8 | Chat1800 | Bm, Tm | 577627 | 151000 | 1780 |
| 9 | Flue1200 | Bm, Tm | 692381 | 197194 | 1235 |
| 10 | Flue1800 | Bm, Tm | 693298 | 197180 | 1720 |
| 11 | Frut1200 | Bm, Tm | 613280 | 157102 | 1210 |
| 12 | Frut1800 | Bm, Tm | 610685 | 155547 | 1780 |
| 13 | Hasl1200 | Rb | 663089 | 174793 | 1165 |
| 14 | Hasl1200 | Bm, Tm | 660291 | 174576 | 1160 |
| 15 | Hasl1800 | Bm, Rb, Tm | 661644 | 175586 | 1760 |
| 16 | Hein1200 | Bm, Tm | 750273 | 179471 | 1190 |
| 17 | Hein1800 | Bm, Tm | 748463 | 177445 | 1790 |
| 18 | Lenk1200 | Bm, Tm | 598900 | 147408 | 1175 |
| 19 | Lenk1800 | Bm, Tm | 597176 | 140747 | 1860 |
| 20 | Lumn1200 | Bm, Tm | 731892 | 173585 | 1190 |
| 21 | Lumn1800 | Bm, Tm | 729458 | 173951 | 1780 |
| 22 | Luet1200 | Rb | 640961 | 166102 | 1200 |
| 23 | Luet1200 | Bm, Tm | 639643 | 165981 | 1210 |
| 24 | Luet1800 | Bm, Rb, Tm | 640773 | 166967 | 1780 |
| 25 | Matt1200 | Rb | 627639 | 126764 | 1095 |
| 26 | Matt1800 | Rb | 637356 | 122106 | 1855 |
| 27 | Ober1200 | Bm, Tm | 597258 | 167136 | 1110 |
| 28 | Ober1800 | Bm, Tm | 599452 | 169396 | 1760 |
| 29 | Obwa1200 | Rb | 619000 | 129824 | 1230 |
| 30 | Obwa1800 | Rb | 617908 | 131673 | 1800 |
| 31 | Reic1200 | Bm, Tm | 622986 | 158775 | 1220 |
| 32 | Reic1800 | Bm, Tm | 625212 | 159410 | 1840 |
| 33 | Sach1200 | Bm, Tm | 659181 | 186656 | 1180 |
| 34 | Sach1800 | Bm, Tm | 659972 | 186844 | 1720 |
| 35 | Unwa1200 | Rb | 579132 | 96053 | 1275 |
| 36 | Unwa1800 | Rb | 586254 | 112200 | 1765 |
| 37 | Vahe1200 | Rb | 598988 | 117825 | 1200 |
| 38 | Vahe1800 | Rb | 603414 | 108839 | 1785 |
| 39 | Vaet1200 | Bm, Tm | 754408 | 203862 | 1190 |
| 40 | Vaet1800 | Bm, Tm | 753125 | 200301 | 1835 |
|  | | | | | |
| The site name consists of the abbreviation of the region and the approximate altitude of population origin (i.e. low: 1200 m a.s.l. and high: 1800 m a.s.l.). | | | | | |
