# Supplementary material for: Plastic Responses to Elevated Temperature in Low and High Elevation Populations of Three Grassland Species
Source: PLoS One. 2014 Jun 5;9(6):e98677. doi: 10.1371/journal.pone.0098677 (PMC4046993; doi:10.1371/journal.pone.0098677)
Supplement: Table S2 — Means and standard errors (SE) grouped by altitude of population origin and temperature treatment for growth-related traits of Trifolium montanum , Ranunculus bulbosus and Briza media . (DOCX) [file pone.0098677.s002.docx]

Supporting Information for doi:10.1371/journal.pone.0098677

| **Table S2.** Means and standard errors (SE) grouped by altitude of population origin and temperature treatment for growth-related traits of *Trifolium montanum*, *Ranunculus bulbosus* and *Briza media*. | | | | | | | | | | | | | | | | | | |
| --- | --- | --- | --- | --- | --- | --- | --- | --- | --- | --- | --- | --- | --- | --- | --- | --- | --- | --- |
|  |  |  |  | *N*_Pop_ | Biomass (g) | |  | Leaf length (cm) | |  | Growth rate | |  | SLA (cm^2^g^–1^) | |  | Above-ground /  total biomass (%) | |
|  |  |  |  |  | Mean | SE |  | Mean | SE |  | Mean | SE |  | Mean | SE |  | Mean | SE |
|  | |  |  |  |  |  |  |  |  |  |  |  |  |  |  |  |  |  |
| *T. montanum* | | |  |  |  |  |  |  |  |  |  |  |  |  |  |  |  |  |
|  | Orig1800 | | Ambient (T_min_) | 10 | 1.71 | 0.15 |  | 6.32 | 0.41 |  | –1.28 | 0.45 |  | 236.39 | 13.01 |  | 34.77 | 1.19 |
|  |  | | Elevated (T) | 10 | 2.64 | 0.11 |  | 5.39 | 0.27 |  | 0.79 | 1.53 |  | 269.37 | 25.89 |  | 31.44 | 1.30 |
|  | Orig1200 | | Ambient (T) | 10 | 3.15 | 0.21 |  | 6.30 | 0.42 |  | –2.24 | 0.62 |  | 252.74 | 17.17 |  | 33.92 | 1.45 |
|  |  | | Elevated (T_plus_) | 10 | 4.63 | 0.23 |  | 10.58 | 0.28 |  | 1.13 | 1.00 |  | 304.30 | 74.74 |  | 30.66 | 2.08 |
| *R. bulbosus* | | |  |  |  |  |  |  |  |  |  |  |  |  |  |  |  |  |
|  | Orig1800 | | Ambient (T_min_) | 7 | 2.08 | 0.16 |  | 2.31 | 0.28 |  | –7.28 | 0.57 |  | 122.83 | 4.48 |  | 40.97 | 4.42 |
|  |  | | Elevated (T) | 7 | 1.80 | 0.18 |  | 2.89 | 0.26 |  | –3.26 | 1.04 |  | 108.53 | 3.31 |  | 38.97 | 4.74 |
|  | Orig1200 | | Ambient (T) | 7 | 2.12 | 0.13 |  | 3.46 | 0.08 |  | –2.70 | 1.24 |  | 141.81 | 3.95 |  | 37.77 | 5.31 |
|  |  | | Elevated (T_plus_) | 7 | 1.98 | 0.15 |  | 4.19 | 0.12 |  | 2.35 | 0.99 |  | 174.19 | 12.30 |  | 33.29 | 4.58 |
| *B. media* | | |  |  |  |  |  |  |  |  |  |  |  |  |  |  |  |  |
|  | Orig1800 | | Ambient (T_min_) | 13 | 1.04 | 0.03 |  | 12.54 | 0.26 |  | 3.45 | 0.36 |  | 213.64 | 4.11 |  | - | - |
|  |  | | Elevated (T) | 13 | 1.20 | 0.03 |  | 11.04 | 0.13 |  | 4.94 | 0.30 |  | 186.55 | 8.89 |  | - | - |
|  | Orig1200 | | Ambient (T) | 13 | 1.17 | 0.02 |  | 10.65 | 0.15 |  | 4.15 | 0.28 |  | 180.57 | 4.90 |  | - | - |
|  |  | | Elevated (T_plus_) | 13 | 1.47 | 0.02 |  | 13.34 | 0.14 |  | 5.36 | 0.47 |  | 200.36 | 5.86 |  | - | - |
|  |  | |  |  |  |  |  |  |  |  |  |  |  |  |  |  |  |  |
|  | | | | | | | | | | | | | | | | | | |
| Orig1800, high elevation plants (1800 m a.s.l.); Orig1200, low elevation plants (1200 m a.s.l.); *N*_Pop_, number of populations. Ambient/Elevated, ambient and elevated temperature treatment respectively. For explanations of the plant traits, see text. Means and standard errors are based on population means. | | | | | | | | | | | | | | | | | | |
