# Supplementary material for: Plastic Responses to Elevated Temperature in Low and High Elevation Populations of Three Grassland Species
Source: PLoS One. 2014 Jun 5;9(6):e98677. doi: 10.1371/journal.pone.0098677 (PMC4046993; doi:10.1371/journal.pone.0098677)
Supplement: Table S3 — Means and standard errors (SE) grouped by altitude of population origin and temperature treatment for reproductive and phenological traits of Trifolium montanum and Ranunculus bulbosus . (DOCX) [file pone.0098677.s003.docx]

Supporting Information for doi:10.1371/journal.pone.0098677

| **Table S3.** Means and standard errors (SE) grouped by altitude of population origin and temperature treatment for reproductive and phenological traits of *Trifolium montanum* and *Ranunculus bulbosus*. | | | | | | | | | | | | | | |
| --- | --- | --- | --- | --- | --- | --- | --- | --- | --- | --- | --- | --- | --- | --- |
|  |  |  |  | Number of flowers | |  | Reproductive allocation (%) | |  | Budding  start (JD) | |  | Flowering  start (JD) | |
|  |  |  |  | Mean | SE |  | Mean | SE |  | Mean | SE |  | Mean | SE |
|  | |  |  |  |  |  |  |  |  |  |  |  |  |  |
| *T. montanum* | | |  |  |  |  |  |  |  |  |  |  |  |  |
|  | Orig1800 | | Ambient (T_min_) | 4.08 | 0.71 |  | 25.94 | 3.36 |  | 180.76 | 4.46 |  | 203.44 | 2.95 |
|  |  | | Elevated (T) | 5.51 | 0.44 |  | 27.17 | 2.67 |  | 167.87 | 4.74 |  | 179.72 | 2.76 |
|  | Orig1200 | | Ambient (T) | 7.74 | 1.40 |  | 35.10 | 3.06 |  | 170.51 | 4.07 |  | 183.43 | 2.79 |
|  |  | | Elevated (T_plus_) | 7.31 | 1.08 |  | 36.75 | 2.91 |  | 150.75 | 2.95 |  | 166.20 | 2.42 |
| *R. bulbosus* | | |  |  |  |  |  |  |  |  |  |  |  |  |
|  | Orig1800 | | Ambient (T_min_) | 6.09 | 0.60 |  | 54.87 | 2.86 |  | 160.23 | 2.02 |  | 179.25 | 1.94 |
|  |  | | Elevated (T) | 4.04 | 0.52 |  | 46.46 | 1.70 |  | 155.04 | 1.63 |  | 170.65 | 0.88 |
|  | Orig1200 | | Ambient (T) | 4.71 | 0.52 |  | 43.75 | 3.25 |  | 151.95 | 3.47 |  | 164.79 | 2.88 |
|  |  | | Elevated (T_plus_) | 4.40 | 0.76 |  | 41.82 | 1.28 |  | 147.39 | 0.93 |  | 156.86 | 1.78 |
|  |  | |  |  |  |  |  |  |  |  |  |  |  |  |
|  | | | | | | | | | | | | | | |
| Orig1800, high elevation plants (1800 m a.s.l.); Orig1200, low elevation plants (1200 m a.s.l.). Ambient/Elevated, ambient and elevated temperature treatment respectively. For explanations of the plant traits, see text. Means and standard errors are based on population means. For number of populations see Table S2. | | | | | | | | | | | | | | |
